# Improving patient safety by enhancing raising concerns at medical school

**DOI:** 10.1186/s12909-018-1281-4

**Published:** 2018-07-28

**Authors:** Luke Johnson, Natasha Malik, Irene Gafson, Naomi Gostelow, Jayne Kavanagh, Ann Griffin, Faye Gishen

**Affiliations:** 1 University College London Medical School, London, England; 2 Royal Free London NHS Foundation Trust, London, UK

**Keywords:** Raising concerns, Patient safety, Whistleblowing, Curriculum development, Innovation, Medical school

## Abstract

**Background:**

Doctors and medical students have a professional responsibility to raise concerns. Failure to raise concerns may compromise patient safety. It is widely known that medical students frequently encounter unprofessional behaviours in the workplace, but little is known about the barriers to raising concerns amongst medical students. This paper explores these issues and discusses some innovations in the medical undergraduate curriculum, offering a good practice model for other medical and healthcare curricula.

We set out to ascertain the attitudes and experiences of medical students in relation to raising concerns. This data was then used to innovate the raising concerns curriculum, and access to the raising concerns system, in order to fundamentally improve patient safety and experience, as well as the student experience.

**Methods:**

The authors conducted a mixed methods quantitative and qualitative research study. Research was based at a UK medical school and involved data collection using an anonymous, voluntary survey emailed to all medical students (*n* = 363) as well as voluntary attendance focus groups (*n* = 24) recruited by email. Both tools investigated student attitudes towards raising concerns and explored student ideas for solutions to improving the process. The focus group data was thematically analysed by three researchers.

**Results:**

The authors identified five key themes which described medical student attitudes towards raising concerns. This article discusses these themes and the resulting work to enhance medical education within the medical school curriculum.

**Conclusions:**

More research is needed to further address the barriers that medical students find in raising concerns. However, despite being a single study in one UK medical school, the authors propose some changes which they hope may inspire other educators to build upon their raising concerns curricula to foster more transparent undergraduate cultures and ultimately improve patient experience and safety.

**Electronic supplementary material:**

The online version of this article (10.1186/s12909-018-1281-4) contains supplementary material, which is available to authorized users.

## Background

The General Medical Council (GMC), the UK’s governing body for doctors, states that doctors and medical students have a professional responsibility to raise concerns and have issued guidance specific to undergraduate medical education in the UK [1]. Failure to raise concerns has been acknowledged as contributing to healthcare incidents, including that of Mid-Staffordshire in the UK [2]. Here, patient safety was drastically compromised, and details of failings took several years to come to light.

Within the UK’s National Health Service (NHS), there have been reforms to protect those raising concerns or whistleblowing, following the Francis Report [2] and the Freedom to Speak Up Review [3]. However, little has been done to empower and protect medical students in similar situations [4, 5], despite calls from some medical educators for this to happen [6–9]. Medical students may encounter unprofessional behaviours in the workplace [6, 10–19] which may negatively impact patient safety and care [2, 3], alongside student mental health [6, 12, 13, 20], resilience [17, 21] and clinical confidence [21, 22]. Students do not always feel able to address such situations [10, 12, 18, 20, 23] and may accept them as part of the medical culture [15]. These are problems also studied across other healthcare professions including nurses, pharmacists, physiotherapists, and dentists [11]. Challenges in raising concerns have been detailed in multiple countries – including the UK, Australia [6], USA, and Canada [19] – and have been shown to be similar in these different settings. However, little research has been done on the raising concerns systems used by students.

In this paper, the authors will use the University College London Medical School (UCLMS) definition of a concern, “a formal expression of grievance or dissatisfaction by a UCLMS MBBS [undergraduate medical degree] student, pertaining to a problem encountered in the context of their degree and typically these concerns are about individuals: doctors, other healthcare professionals, administrators and tutors” [24].

At UCLMS, until 2017, the raising concerns curriculum was covered as a single lecture and small group session within year four. Students typically raise concerns via the raising concerns portal [25] – a dedicated website, monitored by the medical school’s Quality Assurance Unit. The website includes a raising concerns online form, which is not anonymous, to enable the team to follow-through on concerns and to make comments accountable, with the intention of addressing any compromised patient safety. However, the site makes clear that concerns are dealt with confidentially, and that the names of those raising concerns are not passed on to the individual(s) against whom the concern has been brought.

Following research into the challenges students face in raising concerns, the authors sought to understand medical student attitudes and experiences in raising concerns at UCLMS. The data was used to implement relevant changes within the curriculum.

## Methods

This was a mixed methods study gathering qualitative and quantitative data. An anonymous, voluntary, online survey was emailed to all students, using Likert scales and free text responses. In addition, four focus group were conducted to gather more in-depth qualitative data.

### Survey design

The survey was designed by two members of the research team (LJ, IG). Questions were designed to mirror GMC medical student guidance on raising concerns [1] (see Additional file 1: Appendix 1). Twenty questions examined demographic information, attitudes towards raising concerns, personal experiences of raising concerns, and feedback on the current raising concerns curriculum and portal.

Within the survey, students were presented with eight scenarios based on GMC guidance on when to raise a concern (1] (see Additional file 1: Appendix 1). Of the eight scenarios, five were directly linked to patient safety. The remaining three were indirectly linked – ‘staff or team conduct’, ‘teacher conduct’, and ‘teacher performance’ – because of their effect on morale and their impact on training well-equipped and safe future healthcare professionals. Students were presented with predetermined response options and asked to select all reasons which would deter them from raising a concern.

The survey was sent out via email in May 2016 to all UCLMS medical students (a total of 1980 students) and included details of student support services for any students affected by topics discussed. Qualtrics software was used to analyse questionnaire data [26].

### Focus group design

Focus groups took place in December 2016. A grounded theory qualitative approach was used. Participants were recruited by an email sent to all UCLMS students, and each was given an information sheet explaining the aims of the project and that it was voluntary and anonymous (see Additional file 2: Appendix 2). Participants were also asked to sign a consent form (see Additional file 3: Appendix 3). The questions (see Additional file 4: Appendix 4) were designed by LJ and NM to expand upon the salient results from the survey.

Twenty-four students attended four focus groups containing between three and eight people each. Multiple focus groups helped ensure data triangulation and credibility. Each group lasted approximately one hour and was facilitated by a senior medical student (LJ), as it was felt that a faculty member might bias and inhibit student responses. The facilitating student received training from two experienced team members (NM, NG) through educational resources and a one-to-one mentorship session.

Since UCL has an integrated curriculum, all students have some clinical exposure, which increases as students progress to later years. We therefore decided to divide focus groups into two sections, consisting of students taught primarily through lectures who have less clinical contact (‘early years’ – years one, two and three), and students engaged primarily in the clinical environment (‘later years’ – years four, five and six). Two different focus for each section were undertaken. Focus groups were anonymised, audio recorded and transcribed using the “Way with Words” transcription service [27]. These were checked for accuracy by two team members reading each transcript.

The data was inductively thematically analysed by three team members (LJ, NM, IG). Each of the four transcripts was analysed independently by two researchers to ensure data dependability. After immersion in the data, the three members agreed the thematic codes and coding framework. The four transcripts were re-read again to ensure data confirmability, and to confirm data saturation.

### Ethical considerations

The UCL Research Ethics Committee granted approval for the focus groups and all participants gave written consent (see Additional file 3: Appendix 3). The same committee advised that as the survey was anonymous, voluntary, and had no financial reward for completion, formal ethics approval was not required.

## Results

### Survey findings

**Fig. 1:**
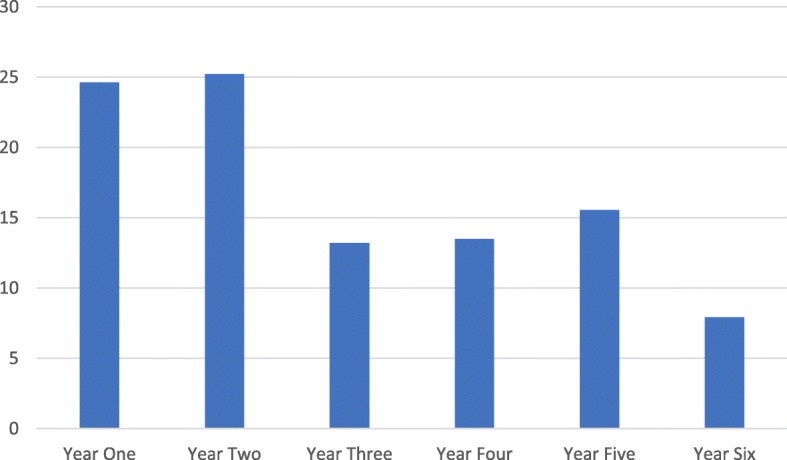
Percentage of total survey respondents from each year. Shows the percentage of respondents from each medical year in the survey questionnaire

Of 1980 students emailed at UCLMS, 363 responded (response rate 18%). Of respondents, 184 (51%) students were female, 151 (42%) students were male, 1 was other, 5 preferred not to say and 22 (6%) did not answer the question on gender. The percentage of total respondents from each year is documented in Fig. 1.

**Fig. 2:**
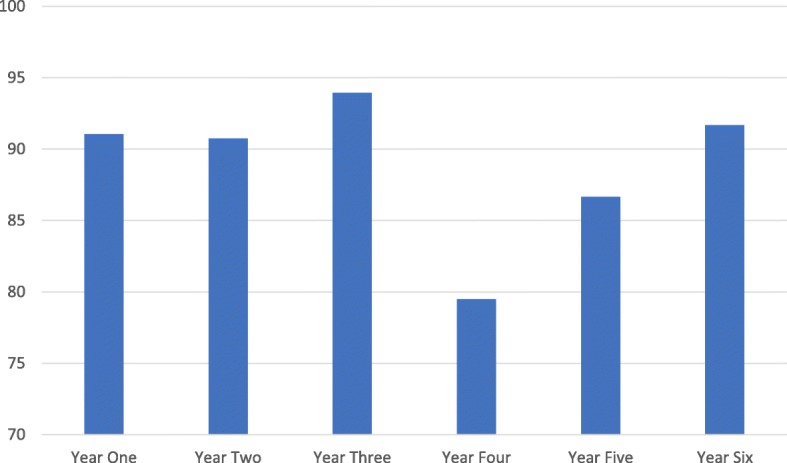
Percentage in each year who strongly agree or agree raising concerns in an important responsibility. Visualises differences within student year groups in strongly ageing or agreeing that raising concerns is an important responsibility

One hundred thirty-one (50%) students strongly agreed that raising concerns was an important responsibility for medical students; 103 (39%) agreed; 25 (10%) somewhat agreed; 1 (0.4%) somewhat disagreed; 3 (1.1%) disagreed; and 0% strongly disagreed. The proportion of students who either strongly agreed or agreed decreased in year 4, the first primarily clinical year, falling from 91% in year 1 to 79% in year 4 (see Fig. 2).

Students felt most sure that they should consider raising a concern for teacher conduct (91%, *n* = 238), staff or team conduct (90%, *n* = 236), and teacher performance (72%, *n* = 189). Students were least sure for issues of staff health (12%, *n* = 30) and staff or team performance (12%, *n* = 31).

**Fig. 3:**
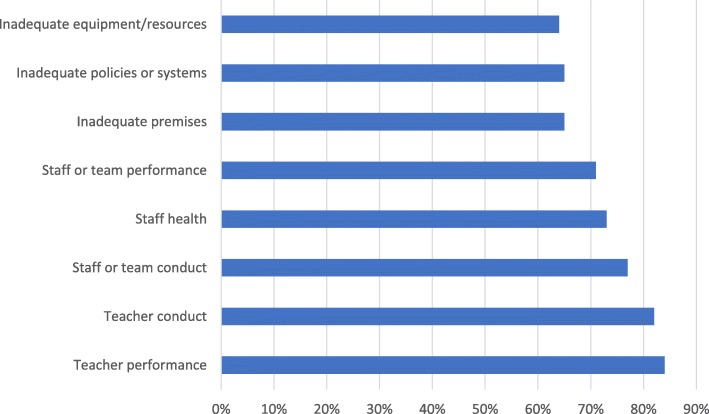
Percentage of students who would raise a concern believing that they should in each situation. Visualises differences in respondents’ willingness to raise a concern in a situation they believe warrants it

Students who believed they should raise a concern were asked if they then would raise a concern in each situation (Fig. 3). This could be an indicator of the ‘conviction’ students have to raise a concern in each situation. Teacher performance (84%, 142 of 169), teacher conduct (82%, 196 of 238) and staff or team conduct (77%, 119 of 168) were the situations most likely to lead to concerns raised; inadequate equipment/resources (64%, 114 of 177), inadequate policies or systems (65%, 114 of 175) and inadequate premises (65%, 102 of 156) were least likely.

Students were asked to select the barriers to raising concerns from a list provided (Table 1). ‘Other’ refers to the free text answers given.

**Table 1:** Deterrents to raising concerns and their frequency

Barrier	Percentage of students that selected this option (*n* = 261)
Belief that nothing would get done	74% (193)
May have a negative impact on your working relationships	63% (165)
May cause problems for colleagues	49% (127)
The situation/occurrence is a one off	46% (119)
May have a negative effect on your career	44% (116)
Don’t know how to raise a concern	41% (107)
May result in a complaint against you	33% (87)
Too much paperwork	18% (46)
Not your responsibility	11% (30)
Other	8% (20)
Nothing would deter me	1% (2)

**Fig. 4:**
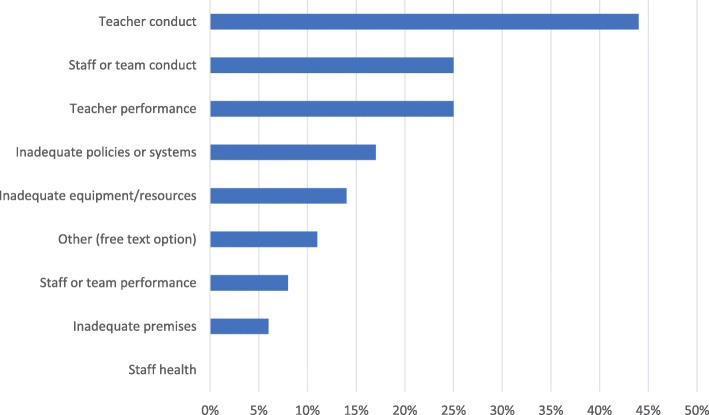
The nature of concerns raised. Visualises the nature of concerns raised for those respondents who had previously used the raising concerns system at the medical school

Students were asked if they had raised concerns before. Those who had were asked how many times they had done so – 20 (56%) responded once, 12 (33%) responded twice, 3 (8%) responded three times, and 1 (3%) responded more than five times. The nature of their concern is outlined in Fig. 4.

Students were also asked if they had ever chosen not to raise a concern - 75 (29%) responded that they had. When asked from the list of barriers detailed in table one what the greatest barrier was to them raising the concern, 43 (60%) responded it was the belief that nothing would get done (futility).

### Focus group findings

Thematic analysis of the focus groups was done through a coding framework (Additional file 5: Appendix 5). This generated five key themes; process of raising concerns, nature of raising concerns, barriers to raising concerns, suggestions for improvement, and parallels to the NHS.

#### Process of raising concerns

This theme incorporated the students’ process of raising concerns, both through formal and informal channels, and their attitudes towards these.

Many would talk to friends or families to look for support or validation of their concern. Students found this helpful in deciding whether to raise a concern more formally:

“I thought it’d be easier…just to talk to my peers before I even thought about escalating” (Focus Group 4, Participant 1).

Students who engaged with the raising concerns system, reported mixed results in outcomes;

“We were a little bit concerned about that particular doctor. And…I think some disciplinary action happened…and so we don’t really see that doctor much anymore” (Focus Group 4, Participant 1).

“We had some issues with the organisation, so we did raise concerns and report them to the division…and then after that we reported it to medical school. But the division didn’t do anything” (Focus Group 4, Participant 1).

Other formal channels for raising concerns, such as the personal tutor system and curriculum feedback questionnaires, were often viewed cynically;

“a lot of them just don’t care…I feel like…this is just an add-on” (Focus Group 4, Participant 2).

#### Nature of concern

This theme incorporated the nature of concerns that students had considered raising or had raised.

There were examples affecting patient safety;

“I was sat in a clinic…it was quite a sensitive consultation and a young girl had come in seeking…a particular medical treatment and it was one she had three times before. And the doctor…really struggled to…not show any sort of judgement for this particular woman…the girl…ran out crying and actually said ‘You’re judging me, you’re judging me!’…I didn’t know what I could’ve said in that situation” (Focus Group 4, Participant 2).

There were comments on breaking confidentiality;

“My friend had their mental health discussed by the tutor with other students when… [he] wasn’t present for reasons of health… you know, he considered it to be confidential” (Focus Group 3, Participant 1).

There were comments about teachers shaming students;

“A senior doctor [was]…delivering teaching…[with] quite a few people…in attendance, and the person I was with was – I mean, I won’t go into too many details, but was just quite humiliated in front of a large group” (Focus Group 3, Participant 1).

There were anecdotes about bullying;

“There were about 15 of us in the room; the doctor consistently picked on this one boy the entire session, for an hour and a half, two hours, and didn’t ask anyone else any questions, just constantly put him on the spot and was quite intimidating” (Focus Group 3, Participant 2).

There were concerns over prejudice, including sexism and racism, including one comment from a teacher;

“Maybe you should consider not doing this specialty because you are a woman” (Focus Group 4, Participant 2).

Students also expressed a variety of concerns about course organisation, personal tutoring and use of questionable or offensive humour.

#### Barriers to raising concerns

This theme incorporated the barriers students felt they faced in raising a concern. On analysis, it became clear that barriers fell into three subthemes (see Additional file 5: Appendix 5):

1. **Comprehension** – understanding when to raise a concern and how to do so
2. **Conviction** – understanding why it is important to raise a concern and recognising the moral responsibility to
3. **Courage** – having the resilience to overcome fear and manage oneself in a situation where a concern is raised

##### Comprehension

The most frequent barrier to emerge was the lack of understanding over what constitutes a valid concern;

“You don’t know like what is like really serious and what isn’t so serious…it’s just really difficult. How are you supposed to know?” (Focus Group 4, Participant 3).

Students were not always sure if they were able to raise concerns on behalf of others.

“The student didn’t want to talk about how they were feeling or they didn’t want to show weakness in front of everyone else, so nobody else stood up and raised a concern on their behalf.” (Focus Group 3, Participant 2).

There was confusion about how to initiate the process;

“I can’t exactly remember how raising a concern works” (Focus Group 4, Participant 1).

##### Conviction

Students struggled to believe that their role in raising concerns is valuable. Some were students who had been disappointed with their past experience of raising concerns.

Some suggested that raising a concern was inconsequential as it was viewed as;

“not going to make a huge difference to your learning” (Focus Group 4, Participant 1).

Some relied on others to raise concerns,

“If there are 340 of you in a lecture, and, you know, you’ve got however many lectures in a day, you just think, well, you know, someone else will probably send that email.” (Focus Group 2, Participant 1).

Feeling part of a culture where medical ‘banter’ was a ‘rite of passage’ was a reason students gave for not raising concerns;

“I think it’s nice to feel like you’re a part of the profession as well; you don’t want to kind of ruin that by, I don’t know, being almost like spying on the doctors or like making them feel like you’re there supervising” (Focus Group 3, Participant 3).

Medical students also experienced doubt over whether their perceptions of events were correct;

“I think you can doubt yourself even if things really aren’t all right” (Focus Group 2, Participant 3).

##### Courage

Fear also played a huge role. To raise a concern was often viewed as brave;

“That we can be quite scared about actually voicing our concerns over such serious problems” (Focus Group 2, Participant 3).

Students worried over repercussions on grades and future career opportunities;

“This is going to affect the rest of my life, my career as a medical professional” (Focus Group 2, Participant 6).

They feared effects of the medical hierarchy and one admitted;

“When you’re a medical student, you feel like it’s not in your place to raise concerns” (Focus Group 2, Participant 5).

There was a perceived peer pressure as well;

“Some of the doctors…who you might want to raise a concern about also have a lot of fans within the student body and there’s plenty of people who will talk really disparaging about people who do raise concerns, because they’re like, oh, they’ve ruined it for the rest of us, now we don’t get teaching with this very clever person.” (Focus Group 3, Participant 3).

Students also reported being afraid of a lack of anonymity or a lack of confidentiality for those who raise concerns.

“I think a lot of the concerns we had about why we didn’t want to raise concerns were to do with anonymity and feeling like it would get revealed who had complained.” (Focus Group 2, Participant 2).

#### Suggestions for improvement

This theme incorporated discussions on how students could be more empowered to raise concerns. Suggestions can be broadly divided into three categories:

1. Improvements in teaching on raising concerns

Suggestions focused on more frequent teaching on raising concerns and teaching earlier within the medical school curriculum;

“If you made it known early…then throughout your medical career you know that there is this well-defined team or contact if you have any sort of issue”. (Focus group 4, Participant 1)

Students commented on wanting more examples of concerns raised in the past;

“It gives people an idea about what might be a valid complaint and how it might be handled and how things would change in the future.” (Focus Group 4, Participant 2)

2. Improvements in the process of raising concerns

Students wanted raising concerns not to be stigmatised within medical school culture. Others liked the idea of an external, impartial department separate from a medical school to deal with concerns raised. One student discussed the possibility of having student ambassadors for raising concerns who would liaise with faculty.

3. Improving support for those who raise concerns

Students wanted the medical school to explicitly let students know they were supporting them in raising concerns;

“Someone high up in the med school who’s going to…say…we’re going to back you up and listen to your concerns”. (Focus Group 2, Participant 4)

#### Parallels to the NHS

This theme incorporates comments made comparing the culture of raising concerns within medical schools with the culture of raising concerns in the NHS.

Participants had preconceptions about the culture of raising concerns in the NHS;

“It should be an open and honest culture, but it really isn’t, and often people aren’t raising concerns” (Focus Group 2, Participant 3).

Hierarchy and repercussions were frequently mentioned. Staff were seen as wanting to keep their head down and just progress through their career and so are;

“scared to out their seniors or their colleagues” (Focus Group 4, Participant 1).

One said;

“If that’s what things are like in the NHS, then why should we complain about it?” (Focus Group 3, Participant 1).

## Discussion

This paper draws parallels with previous papers on healthcare professionals’ attitudes on and experience with raising concerns.

As clinical exposure increases most significantly within the UCLMS curriculum (year four), students appear to experience the greatest drop in belief raising concerns is an important responsibility. It would follow that more clinical exposure leads to more likelihood to encounter a greater variety of the ethical dilemmas highlighted in the literature [6, 10–19]. This drop in belief with greater clinical exposure is something not shown in the literature before. Healthcare educators need to utilise this finding in their own curricula.

A notable minority of students (29%) had chosen not to raise concerns, as shown elsewhere [12]. The reasons behind this are complex and include a combination of the barriers detailed in table one, as well as some expressed within the focus groups – uncertainty of validity of concerns, uncertainty how to raise concerns, fear of repercussion, believed lack of impact, uncertainty of responsibility, and normalisation of unprofessional behaviour to name a few. These barriers are reiterated elsewhere in the literature [10, 12, 15, 18, 20, 23], further emphasising the need for medical educators to address them. Attempting to address and alleviate these barriers is important to creating a culture where students are better empowered to raise concerns, therefore impacting patient safety.

A belief that nothing would get done is by far the most common reasons preventing students from raising concerns.

The authors acknowledge certain limitations. The self-selected volunteers who partook in the survey and focus groups were likely to include those with stronger opinions on raising concerns, whether positive or negative. Further research in to this area could focus on the use of longitudinal data (such as [9]), and include more research into other healthcare learners.

We adapted our curriculum based around these highlighted issues of comprehension, conviction and courage, in order to enhance patient safety.

To address the comprehension, the existing year four session was made more comprehensive, explaining more of when the GMC suggests raising concerns, and explaining the challenges students face to raising concerns. We used results from this research to help explain this and then explained how UCLMS was helping address these challenges. The session was moved to the start of the academic year to enable students to feel better prepared going into the clinical environment.

To address the conviction, and to introduce raising concerns earlier in the curriculum, a tutor-facilitated small group session has now been instituted in year one. The session examines how a lack of effective raising concerns contributed to the Mid-Staffordshire healthcare climate, including patient safety.

To address the courage, a Schwartz Round [28], a large group reflective forum with growing popularity in medical schools [29], was held in 2017 on the topic of ‘Courage’. The forum allowed fifth year students to discuss raising concerns in a confidential and safe space.

## Conclusion

Through a mixed-methods approach, results appeared to indicate that as clinical exposure increases most significantly, students experience the greatest drop in their belief of responsibility to raise concerns. There also appears to be a gap between students’ understanding of when they should raise concerns and when they actually would. Within the five key focus group themes, the subcategories of barriers to raising concerns – comprehension, conviction and courage – allowed us to address these findings within the curriculum.

We hope that our curriculum framework discussed in this article will inspire other healthcare educators to reflect on their curricula. Innovations to empower students in raising concerns could be shared to encourage organisations to learn from each other. The authors hope that their research will help to push this process forward so further improving patient safety across healthcare in the UK and internationally.

## Additional files

Additional file 1: **Appendix 1.** A list of the questions asked in the survey. (DOCX 21 kb)

Additional file 2: **Appendix 2.** The information sheet received by focus group participants to explain the nature of the study and information on the focus groups in which they were invited to partake in. (DOCX 14 kb)

Additional file 3: **Appendix 3.** The consent form received by focus group participants to understand what they were agreeing to by partaking in the focus group. (DOCX 14 kb)

Additional file 4: **Appendix 4.** A list of the questions asked to focus group participants. (DOCX 12 kb)

Additional file 5: **Appendix 5.** The coding framework evolved by the three analysers by the team members involved in the focus group research (LJ, NM, IG). (DOCX 14 kb)

## Abbreviations

GMC: General Medical Council
MBBS: Undergraduate Medical Degree
NHS: National Health Service
UCLMS: University College London Medical School
