# Supplementary material for: Improving patient safety by enhancing raising concerns at medical school
Source: BMC Med Educ. 2018 Jul 28;18:171. doi: 10.1186/s12909-018-1281-4 (PMC6064143; doi:10.1186/s12909-018-1281-4)
Supplement: Supplementary file 1 — Appendix 1. A list of the questions asked in the survey. (DOCX 21 kb) [file 12909_2018_1281_MOESM1_ESM.docx]

1. What is your gender?
   1. Female
   2. Male
   3. I’d rather not say
   4. Other
2. What is your ethnic group? (standard drop down options)
3. Did you have a degree before commencing medical school?
   1. Y
   2. N
4. What year of medical school are you in?
   1. Year 1
   2. Year 2
   3. iBSc
   4. Year 4
   5. Year 5
   6. Year 6
5. How much do you agree with the following statement: ‘Raising concerns is an important responsibility for medical students’ (standard 6 point satisfaction Likert scale)
6. Please look at the GMC’s most common reasons for raising concerns below. Please consider whether you ‘should’ or ‘should not’ raise concerns in these circumstances and select the appropriate option. You may select more than one option.
   1. Inadequate equipment/resources
   2. Inadequate policies or systems
   3. Inadequate premises
   4. Staff health
   5. Staff or team conduct
   6. Staff or team performance
   7. Teacher conduct
   8. Teacher performance
7. Please look at the GMC’s most common reasons for raising concerns below. Please consider whether you ‘would’ or ‘would not’ raise concerns in these circumstances and select the appropriate option. You may select more than one option.
   1. Inadequate equipment/resources
   2. Inadequate policies or systems
   3. Inadequate premises
   4. Staff health
   5. Staff or team conduct
   6. Staff or team performance
   7. Teacher conduct
   8. Teacher performance
8. Which of the following would deter you from raising a concern as a medical student?
   1. Belief that nothing would get done
   2. Don’t know how to raise a concern
   3. May cause problems for colleagues
   4. May have a negative effect on your career
   5. May have a negative effect on your working relationships
   6. May results in a complaint against you
   7. Not your responsibility
   8. Nothing would deter me
   9. The situation/occurrence is a one off
   10. Too much paperwork
   11. Other (please specify)
9. Have you ever informally raised a concern(s) about an incident/event/person at medical school including whilst on clinical placement?
   1. Y
   2. N
10. Have you ever formally raised a concern(s) about an incident/event/person at medical school including whilst on clinical placement?&nbsp;E.g. Used the raising concerns portal, raised a concern through an SEQ (student evaluation questionnaire), approached your module/clinical lead, or reported directly to the GMC.
    1. Y
    2. N
    3. I’d rather not say
11. How many times have you raised a concern during medical school?
    1. Once
    2. Twice
    3. Three times
    4. Four times
    5. Five times
    6. More than five times
12. If Y to 11, what was the nature of your concern? (options as per Qu. 7 + other).
13. What was your overall satisfaction with the way in which it was investigated/dealt with?

If you have raised more than one concern, you can select more than one box if your satisfaction differed. (standard 6 point Likert satisfaction scale)

1. Have you ever chosen not to formally raise a concern(s) about an incident/event/person at medical school including whilst on clinical placement?
   1. Y
   2. N
   3. I’d rather not say
2. If Y to 14, What was the nature of your concerns(s)? You may select more than one option (options as per Qu. 7 + other).
3. Which of the following deterred you the most? You may select more than one option. (options as per Qu. 8).
4. Please feel free to provide additional information to explain your reasons for not raising a concern in the past.
5. What forms of teaching have you received about raising concerns? You may select more than one option.
   1. Lecture
   2. Role modelling
   3. Small group teaching
   4. Clinical placements
   5. None
   6. Other
6. To what degree do you agree with the following statements: (Standard 6 point Agreement Likert scale)
   1. The teaching I have received about raising concerns has adequately prepared me to raise concerns as a medical student
   2. The teaching I have received about raising concerns has adequately prepared me to raise concerns as a doctor
7. What do you think medical schools could do to facilitate raising concerns within medical education? (free text box)
