# Supplementary material for: Improving patient safety by enhancing raising concerns at medical school
Source: BMC Med Educ. 2018 Jul 28;18:171. doi: 10.1186/s12909-018-1281-4 (PMC6064143; doi:10.1186/s12909-018-1281-4)
Supplement: Supplementary file 2 — Appendix 2. The information sheet received by focus group participants to explain the nature of the study and information on the focus groups in which they were invited to partake in. (DOCX 14 kb) [file 12909_2018_1281_MOESM2_ESM.docx]

| **Information sheet**  **You will be given a copy of this information sheet.**  Title of Project: **Understanding medical students’ approaches to raising concerns**  This study has been approved by the UCL Research Ethics Committee (Project ID Number): 8027/001 | |
| --- | --- |
| Name | Luke Johnson |
| Work Address | UCL Medical School |
| Contact Details | luke.johnson.11@ucl.ac.uk |
| We would like to invite all medical students to participate in this research project.  **Details of Study:**  The Project has two aims - firstly, to get a better understanding of the problem of raising concerns for UCL medical students in ethically-compromised incidents; and secondly, to develop novel ways of addressing these problems with a view to better equipping medical students to raise concerns in future incidents.  As part of the project, we hope to recruit groups of UCL medical students to discuss your thoughts on raising concerns as medical students. This will involve discussion around if medical students have a responsibility to raise concerns, your thoughts on the culture of raising concerns, sharing in detail experiences of when you have chosen to raise concerns or chosen not to raise concerns and why, and what, if anything, potentially needs to change to make medical students more prepared to raise concerns. Evidently, the material above is particularly sensitive and you will be asked to anonymise any anecdotes shared involving students or doctors not present and on keeping confidential all material discussed within the session private – not discussing it with other students, friends or family members. You will be required to sign a form before the discussion to agree to this.  Groups will contain 8 medical students and comprise of either pre-clinical or clinical medical students – years 1, 2 and 3, and years 4,5 and 6 respectively. There will also be 1 group facilitator, and 1 supervisor. The discussion will last approximately one hour and will take place in a prebooked UCL meeting room which you will be emailed about. Refreshments will be available.  Please ask us if there is anything that is not clear or if you would like more information.  It is up to you to decide whether to take part or not; choosing not to take part will not disadvantage you in any way. If you do decide to take part you are still free to withdraw at any time – either before the session or within the session itself – and without giving a reason.  All data used will be anonymised and, where necessary, adapted to make it more general and therefore untraceable to a person. Data collected will be used in anticipated academic publications hoping to detail the challenges faced by medical students on raising concerns and what can be done to address these challenges. You will be notified of any publications which come out of this piece of work. Data may also be shared in academic presentations relating to the challenges of raising concerns amongst medical students as well.  **All data will be collected and stored in accordance with the Data Protection Act 1998.** Data will only be accessed by Luke Johnson, who is leading this project, and relevant members of the UCL medical school staff. Data will be only accessed for gathering information relating to raising concerns research work. Recorded interviews will be written up and then the recording will be deleted.  **Thank you for reading this information sheet and for considering take part in this research.** | |
