# Supplementary material for: Improving patient safety by enhancing raising concerns at medical school
Source: BMC Med Educ. 2018 Jul 28;18:171. doi: 10.1186/s12909-018-1281-4 (PMC6064143; doi:10.1186/s12909-018-1281-4)
Supplement: Supplementary file 3 — Appendix 3. The consent form received by focus group participants to understand what they were agreeing to by partaking in the focus group. (DOCX 14 kb) [file 12909_2018_1281_MOESM3_ESM.docx]

| Consent form    Please complete this form after you have read the Information Sheet and/or listened to an explanation about the research.  Title of Project: **Understanding medical students' approaches to raising concerns**  This study has been approved by the UCL Research Ethics Committee (Project ID Number): 8027/001  Thank you for your interest in taking part in this research. Before you agree to take part, the person organising the research must explain the project to you.  If you have any questions arising from the Information Sheet or explanation already given to you, please ask the researcher before you to decide whether to join in. You will be given a copy of this Consent Form to keep and refer to at any time. |
| --- |
| **Participant’s Statement**  I   - Have read the notes written above and the Information Sheet, and understand what the study involves. - Understand that if I decide at any time that I no longer wish to take part in this project, I can notify the researchers involved and withdraw immediately. - Consent to the processing of my personal information for the purposes of this research study. - Understand that such information will be treated as strictly confidential and handled in accordance with the provisions of the Data Protection Act 1998. - Agree that the research project named above has been explained to me to my satisfaction and I agree to take part in this study. - I understand my participation will be recorded and I consent to the use of this material as part of the project. - Agree to anonymise any information I share here today which involves other people, including students and doctors. - Agree not to share information discussed today with others afterwards – whether students, friends, family members or other people. - I understand that the information I have submitted will be published as a report and I will be sent a copy. Confidentiality and anonymity will be maintained and it will not be possible to identify me from any publications. |
| Signed: Date: |
