# Supplementary material for: Improving patient safety by enhancing raising concerns at medical school
Source: BMC Med Educ. 2018 Jul 28;18:171. doi: 10.1186/s12909-018-1281-4 (PMC6064143; doi:10.1186/s12909-018-1281-4)
Supplement: Supplementary file 4 — Appendix 4. A list of the questions asked to focus group participants. (DOCX 12 kb) [file 12909_2018_1281_MOESM4_ESM.docx]

**1**) Do you have any experience of raising concerns? This can be either you, or others you know.

a.       What was the nature of the incident? (keeping all names anonymous)

b. Why did you decide to raise your concern?

c.      Who did you raise it to? How did you raise it? How was it dealt with? Were you satisfied with the way it was dealt with?

**2**) What, if anything, would stop you from raising a concern?

a. How could these concerns be alleviated?

**3**) When faced with an incident, have you ever chosen not to raise a concern, or has this happened to someone that you know?

a.      What was the nature of the incident?

b.      Why did you decide not raise the concern?

**4**) What do you think the culture of raising concerns is like among medical students?

**5)** What do you think about the culture of raising concerns in the NHS?

**6**) What, if anything, potentially needs to change to make medical students more prepared to raise concerns? Who needs to be involved in changing this? What role should the medical school play in changing this?

**7**) What for you has been the most important point made in this discussion on raising concerns?
