# Supplementary material for: Improving patient safety by enhancing raising concerns at medical school
Source: BMC Med Educ. 2018 Jul 28;18:171. doi: 10.1186/s12909-018-1281-4 (PMC6064143; doi:10.1186/s12909-018-1281-4)
Supplement: Supplementary file 5 — Appendix 5. The coding framework evolved by the three analysers by the team members involved in the focus group research (LJ, NM, IG). (DOCX 14 kb) [file 12909_2018_1281_MOESM5_ESM.docx]

_sBARRIERS_

**_Comprehension_**

_-clarity of process_

_-length of process_

_-repercussions (for others)_

_-happened to someone else_

_-personal/professional blurred line_

_-insufficient information known about event_

**_Conviction_**

_- Self-doubt/blame_

_-personal responsibility (only self-involved)_

_-not a priority_

_-camaraderie_

_-"others put up with it"_

_-patient safety issue_

_- Disillusionment of efficacy_

_-isolated event_

**_Courage_**

_-fear/rumours_

_-reputation (negative impact on you, "feel ridiculous", implicate others)_

_-hierarchy_

_-confidentiality and anonymity_

_-peer pressure_

_- support_

_PROCESS_

_-action taken_

_-directly approached individual_

_-personal tutors_

_-friends/family_

_-CPP tutors_

_-SEQs_

_-module reps_

_-educate students_

_- Do nothing_

_TYPE_

_-Ism's_

_-personal tutor issues_

_-Unprofessional behaviour_

_-Black humour_

_-Biased information_

_-Affecting someone else_

_-Teaching_

_-Confidentiality breach_

_-Bullying/harassment_

_NHS PARALLELS_

_SUGGESTIONS FOR IMPROVEMENT_

_-teaching/education (cover earlier in medical school, cover more, educate doctors)_

_-process (raising concerns policy, handbook, SEQs, confidentiality, anonymity, feedback of changes, rep issues)_

_-support (personal approach, personal tutor)_
